# Biological tumor volume predicts survival in recurrent High-Grade glioma: A multiparametric [^18^F]FET PET/MRI study

**DOI:** 10.1007/s00259-025-07469-8

**Published:** 2025-07-24

**Authors:** Dylan Henssen, Michael Rullmann, Anne I.J. Arens, Andreas Schildan, Stephan Striepe, Matti Schürer, Cordula Scherlach, Katja Jähne, Ruth Stassart, Osama Sabri, Clemens Seidel, Swen Hesse

**Affiliations:** 1 Department of Nuclear Medicine, University Hospital Leipzig, Leipzig, Germany; 2 Department of Medical Imaging, Radboud University Medical Center, Nijmegen, The Netherlands; 3 Institute for Neuroradiology, University Hospital Leipzig, Leipzig, Germany; 4 Department of Neurosurgery, University Hospital Leipzig, Leipzig, Germany; 5 Institute of Neuropathology, University of Leipzig, Leipzig, Germany; 6 Department of Radiation Oncology, University Hospital Leipzig, Leipzig, Germany

**Keywords:** High-grade glioma, Tumor recurrence, Overall survival, [¹⁸F]FET PET/MRI, Biological tumor volume

## Abstract

**Background and purpose:**

Single-session, multiparametric [¹⁸F]FET PET/MRI is used to detect tumor recurrence in high-grade glioma, but its prognostic value for overall survival remains uncertain. This study evaluated whether biological tumor volume, tumor-to-background ratio (TBRmax), cerebral blood volume (rCBVmax), and choline/NAA ratio (Cho/NAA) could predict survival in recurrent high-grade glioma.

**Materials and methods:**

Twenty-six patients with histopathologically confirmed tumor progression underwent simultaneous [¹⁸F]FET PET/MRI. PET-derived biological tumor volume and TBRmax, MRI-derived rCBVmax, and Cho/NAA ratio were analyzed. A Cox proportional hazards model assessed associations with overall survival, adjusting for the number of lesions and treatment strategy.

**Results:**

Biological tumor volume (hazard ratio = 2.22, 95%-CI: 1.035–4.762, *p* = 0.041) and the number of lesions (hazard ratio = 1.03, 95%-CI 1.00–1.06, *p* = 0.036) were significantly associated with survival. TBRmax (*p* = 0.089), rCBVmax (*p* = 0.088), and Cho/NAA ratio (*p* = 0.734) were not predictive. Treatment strategy after tumor recurrence diagnosis did not significantly impact overall-survival (HR = 0.208, *p* = 0.649). PET/MRI interaction terms did not enhance survival prediction.

**Conclusion:**

Biological tumor volume is a significant prognostic imaging biomarker in recurrent high-grade glioma, emphasizing tumor burden over metabolic activity or perfusion of individual lesions. Volume-based PET metrics may offer better survival prediction than traditional PET or MRI parameters. Prospective multicenter studies are needed to validate these findings and explore automated segmentation and machine learning approaches for improved prognostication.

**Supplementary Information:**

The online version contains supplementary material available at 10.1007/s00259-025-07469-8.

## Introduction

Tumor recurrence is often encountered in diffuse infiltrating adult type gliomas (i.e., oligodendroglioma grade 3, astrocytoma grade 3 and 4, glioblastoma [1]) after treatment with surgical resection and postoperative chemoradiotherapy. Tumor recurrence lesions in high-grade gliomas are visible on magnetic resonance imaging (MRI) as new contrast-enhancing lesions within or outside the radiation field which are surrounded by an increasing region of T2w and FLAIR hyperintensity [2, 3]. Tumor recurrence lesions show an increased relative cerebral blood volume (rCBV) on dynamic susceptibility contrast perfusion weighted imaging (DSC-PWI), reflecting their neo-angiogenesis properties [4, 5]. Proton Magnetic Resonance Spectroscopy (^1^H MRS) can help to measure the increased cellular turnover (i.e., an increased choline (Cho) peak at 3.2 ppm) and a decreased neuronal density (i.e., a decreased N-acetylaspartate (NAA) peak at 2.0 ppm) [5, 6]. Furthermore, positron-emission tomography (PET) imaging by use of O-(2-[^18^F]fluoroethyl)-L-tyrosine ([^18^F]FET), a radio-labeled amino-acid, shows an increased amino-acid transport in tumor recurrence lesions [7, 8]. To this end, [^18^F]FET PET/MRI has been proposed the most optimal imaging method to diagnose tumor recurrence and discern it from treatment related abnormalities (i.e., pseudoprogression and radiation necrosis) [9, 10], an entity with similar imaging features on conventional MRI sequences [2, 3]. Although the diagnostic properties of [^18^F]FET PET/MRI for the evaluation of lesions suspected for tumor progression are well-established, it remains partially unknown whether this information on biological and structural properties of the recurrent lesions are predictive of overall-survival in high-grade gliomas with tumor progression. Various studies have shown that biological tumor volume in the pretreatment or posttreatment setting has prognostic value for predicting progression-free survival and overall-survival in glioma patients [11–14]. MRI-based studies provided promising results that (micro)structural properties of tumor progression lesions are relevant for overall-survival prediction [15–17]. Only one study combined the particular strengths of PET/MRI in post-treatment glioma imaging, showing that functional connectivity between recurrent tumor locations and resting-state networks may serve as an additional imaging biomarker to predict overall-survival [18]. However, the number of studies investigating the clinical value multiparametric [^18^F]FET PET/MRI needs to increase to demonstrate its clinical value and ultimately lead to the inclusion of quantitative analysis of multiparametric [^18^F]FET PET/MRI data in consensus guidelines and recommendations.

To foster this clinical translation of multiparametric [^18^F]FET PET/MRI and quantitative analysis, this study investigated the predictive value of increased amino-acid transport, rCBV-values and the ^1^H MRS spectrum, derived from a single-session, multiparametric [^18^F]FET PET/MRI examination, in recurrent high-grade glioma patients.

## Materials and methods

### Ethical assessment

The local medical ethical committee approved the conduction of this study at our hospital (ethical review board assigned file number: 014/21-ek).

### Radiosynthesis of [^18^F]FET

**Fig. 1:**
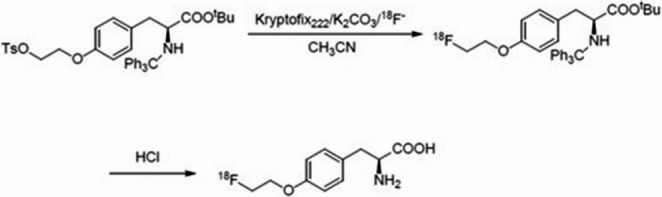
Radiosynthesis of [^18^F]FET by nucleophilic substitution

The [^18^F]FET production process was a two-step labeling/deprotection reaction on an AllinOne synthesis module from Trasis (Ans, Belgium; https://www.trasis.com/en/product/fet) and followed an original protocol from Trasis (Fig. 1). [^18^F]Fluoride was eluted from an anion exchange resin using a cryptofix/water/acetonitrile solution. The eluate was then evaporated to dryness. Subsequently, the tosylate precursor (TET or (2 S)-O-(2’-tosyloxyethyl)-N-trityl-tyrosine-tert-butyl ester) was added. The labeling reaction was carried out at a temperature of 120 °C for a duration of ten minutes. The protective groups were then cleaved off by acid hydrolysis at 110 °C for a duration of ten minutes. The product was purified by means of solid phase extraction, formulated in citrate buffer, and filtered through a 0.22 μm sterile filter prior to dispensing.

### Study cohort and imaging protocol

**Fig. 2:**
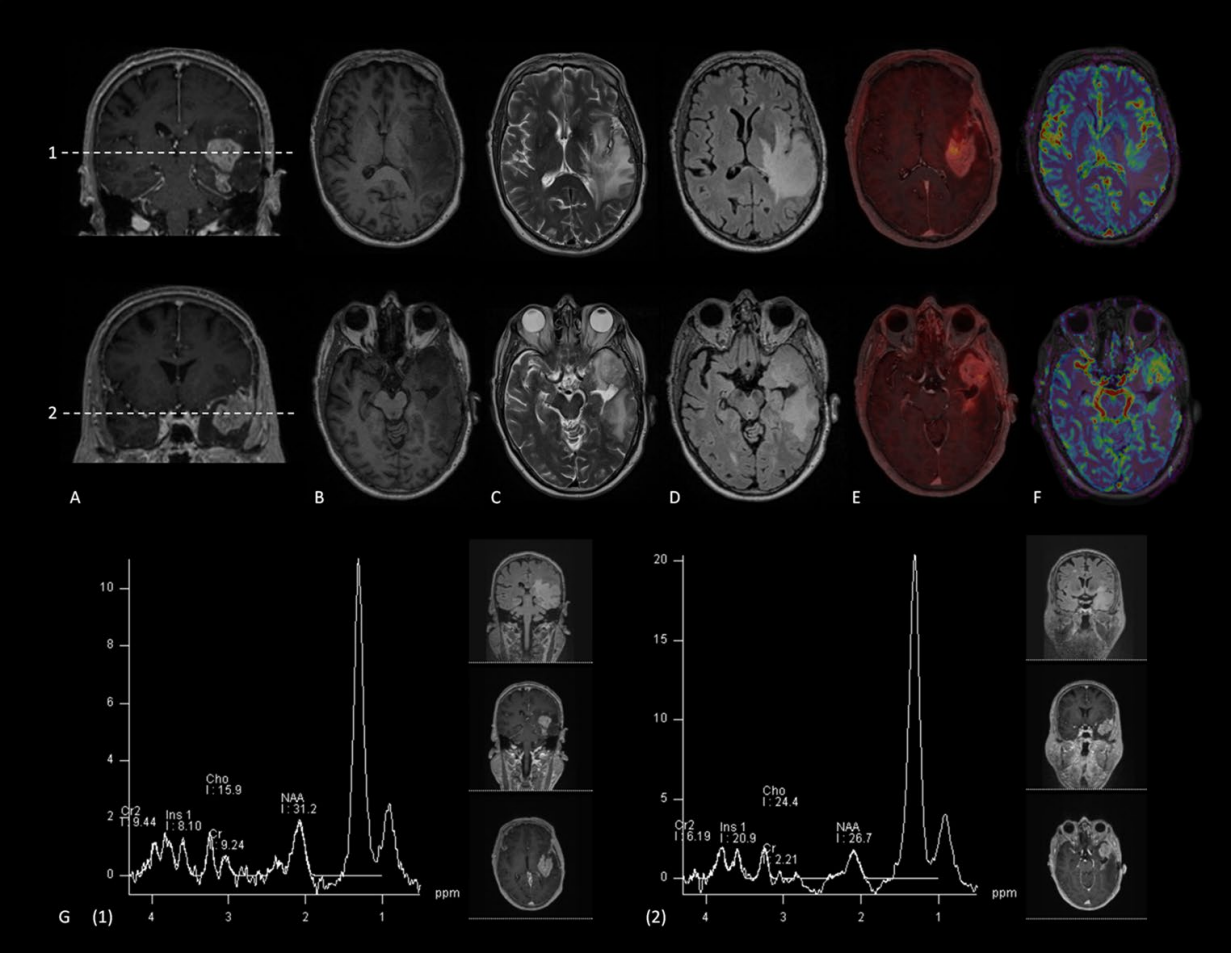
Representative [^18^F]FET PET/MRI of a 75 year old glioblastoma patient with two tumor localizations within the left hemisphere. A: Coronal T1-weighted, post-contrast images showing the tumor localizations in the left hemisphere. The dashed line provides the level of the transverse images (panel B-F); B: T1-weighted, pre-contrast images; C: T2-weighted images; D: FLAIR images; E: [18F]FET PET data fused with transverse T1-weighted, post-contrast images; F: rCBV-maps derived from DSC-PWI; G: MRS spectra of the two tumor localizations. One contrast-enhancing mass, surrounded by perilesional T2-FLAIR hyperintensities, is located within the left anterior temporal lobe. The lesion shows a pathologically increased amino-acid transport, hyperperfusion, local tissue disintegration (reflected by an increased Cho-peak at 3.2 ppm) and decreased neuronal density (reflected by a decreased NAA-peak at 2.0 ppm) The second contrast-enhancing mass, surrounded by perilesional T2-FLAIR hyperintensities, is located in the left operculum region. The lesion shows a pathologically increased amino-acid transport, moderately increased local perfusion, pronounced local tissue disintegration (reflected by an increased Cho-peak at 3.2 ppm) and considerably decreased neuronal density (reflected by a decreased NAA-peak at 2.0 ppm)

Patients aged 18 years or older with new contrast-enhancing lesions after treatment for high-grade glioma, histopathological confirmed as tumor progression, were eligible for inclusion in this study. Patients who declined to share their anonymous data for scientific purposes were excluded from the study. All imaging data were acquired on a hybrid 3 T PET/MR system (Biograph mMR, Siemens Healthineers, Erlangen, Germany). At the time of intravenous bolus injection of 207 ± 41 MBq [^18^F]FET, dynamic brain PET data were acquired in 3D list-mode over 60 min, and reconstructed into a 256 × 256 matrix (voxel size: 1.00 × 1.00 × 2.03 mm³) employing the built-in ordered subset expectation maximization algorithm with 8 iterations, 21 subsets and a 3 mm Gaussian filter. For attenuation correction, the vendor-provided HiRes method was used, which combines the individual Dixon attenuation correction approach with a bone attenuation template. All MRI data were acquired simultaneously to the PET data. MRI sequences included T1w, T2w, FLAIR, T1w after administration of intravenous Gadolinium-based contrast agents, DSC-PWI, and single-voxel ^1^H MRS (more information on the specific parameters per pulse sequence can be found in Supplementary Table 1) (Fig. 2).

Voxel placement was carried out by the technicians, covering the contrast-enhancing lesions. If multiple, separate contrast-enhancing lesions were visible, multiple voxels were placed for ^1^H MRS measurement in each voxel. When in doubt, a nuclear medicine physician was available to help place the single-voxel. Analysis of the DSC-PWI data, a workstation equipped with Syngo.via VB60 (Siemens Healthineers, Erlangen, Germany) was used. A region of interest (ROI) was manually placed by a radiologist-nuclear medicine physician (DH; 10 years of experience with (experimental) neuro-imaging) to contain the contrast-enhancing lesion and blinded for the patient OS. The contralateral homonymous region was used for in-subject normalization to calculate the rCBVmax-value. The radiologist-nuclear medicine physician ensured that no large blood vessels or parts of the choroid plexus were included in the ROI. If multiple, separate contrast-enhancing lesions were visible, multiple ROIs were placed. Spectral fitting of the ¹H MRS data was performed using Siemens syngo.MR Spectroscopy software (Siemens Healthineers, Erlangen, Germany). These reconstructions were used to calculate the Cho/NAA-ratio for each lesion.

Dynamic PET data were motion-corrected and co-registered with the individual MRI images using PMOD (PMOD Technologies LLC, Zurich, Switzerland). Static PET images were generated for the fixed time interval 20–40 min post-injection. These static reconstructions were used for tumor-to-brain ratio (TBR) calculations. Within-subject normalization was performed by assessing the background activity in each patient. Background activity was measured by placing an ROI in the contralateral hemisphere covering both white and gray matter [19, 20], which is in line with the German guideline for amino-acid imaging [14]. Another ROI was placed covering each of the contrast-enhancing lesions. All ROIs were manually placed by a radiologist-nuclear medicine physician (DH). The biological tumor volume was assessed by semi-automatic segmentations of the regions with increased amino acid transport. Volumes of interest (VOIs) were semi-automatically delineated using the 3D iso-contouring approach based on pixel intensity thresholds in PMOD (PMOD Technologies LLC, Zurich, Switzerland). The biological tumor volume reflected the volume of tissue with increased amino acid transport, defined as areas showing increased [18 F]FET uptake, using a literature-based uptake threshold of a tumor-to-background ratio (TBR) ≥ 1.6 relative to the mean background activity [21, 22].

Overall-survival after PET imaging was derived from the electronic patient file. Other data that were derived from the electronic patient file included gender, age, histopathological diagnosis and treatment strategy after tumor recurrence diagnosis.

### Statistical analysis

A Cox proportional hazards regression model was used to assess the relationship between imaging-derived parameters and overall-survival in patients with histopathologically confirmed tumor progression of high-grade glioma. The dependent variable was overall-survival, defined as the time from PET/MRI acquisition to death (in days). Patients who were alive at the last follow-up were censored. Independent variables included TBRmax-values derived from static [^18^F]FET PET imaging, rCBVmax-values obtained from DSC-PWI, and the Cho/NAA ratio from ¹H MRS. Additionally, the number of contrast-enhancing lesions and the biological tumor volume were included as a covariate to account for tumor burden. The proportional hazards assumption was evaluated using log-log plots. A stepwise approach was used to identify significant predictors, with an initial significance level set at *p* < 0.05. To assess multicollinearity among the independent variables included in the Cox proportional hazards regression model, linear regression collinearity diagnostics were performed. All predictors of the biological-clinical model were simultaneously entered as independent variables in a linear regression model, and collinearity statistics (tolerance and variance inflation factor (VIF)) were calculated. Multicollinearity was considered acceptable if VIF values remained below 2.0. The same methodology was followed to assess the multicollinearity of the combined PET/MRI predictors (e.g., rCBV x TBRmax, Cho/NAA ratio x rCBV). All statistical analyses were performed using SPSS (version 29, IBM Corp., Armonk, NY, USA) and R software package [23].

## Results

Twenty-six patients suffering from tumor recurrence were included in this study (55 ± 13 years, 11 females). Sixteen patients had a primary diagnosis of glioblastoma, two patients had a primary diagnosis of oligodendroglioma grade 3 and six patients suffered from a recurrent astrocytoma (four grade 3 astrocytomas; two grade 4 astrocytomas). Fourteen patients were deceased at last follow-up. A Kaplan-Meier curve is presented in Fig. 1. Median overall-survival of the deceased patients after the multiparametric [^18^F]FET PET/MRI was 238 days, ranging from 118 to 973 days. Recurrent glioblastoma patients had a similar overall-survival than patients who suffered from a recurrent oligodendroglioma or astrocytoma (*p* = 0.304) (Table 1).

**Table 1:** Overview of characteristics of patients included in this study

Model Fit Statistics		Value	*p*-value
−2 Log Likelihood (Null Model)		68.919	N/A
−2 Log Likelihood (Full Model)		58.306	N/A
Omnibus Chi-square		12.071	0.002
Predictor	**HR (Exp(B))**	**95% CI**	**p-value**
Number of lesions	2.220	1.035–4.762	0.041
Biological tumor volume	1.032	1.002–1.064	0.036
Model Fit Statistics		**Value**	**p-value**
−2 Log Likelihood (Null Model)		67.836	N/A
−2 Log Likelihood (Full Model)		61.476	N/A
Omnibus Chi-square		6.031	0.110
Predictor	**HR (Exp(B))**	**95% CI**	**p-value**
TBRmax	1.606	0.931–2.769	0.089
rCBVmax	0.278	0.064–1.208	0.088
Cho/NAA-ratio	0.994	0.963–1.027	0.734
Treatment strategy	0.208	0.042–1.042	0.649

**Fig. 3:**
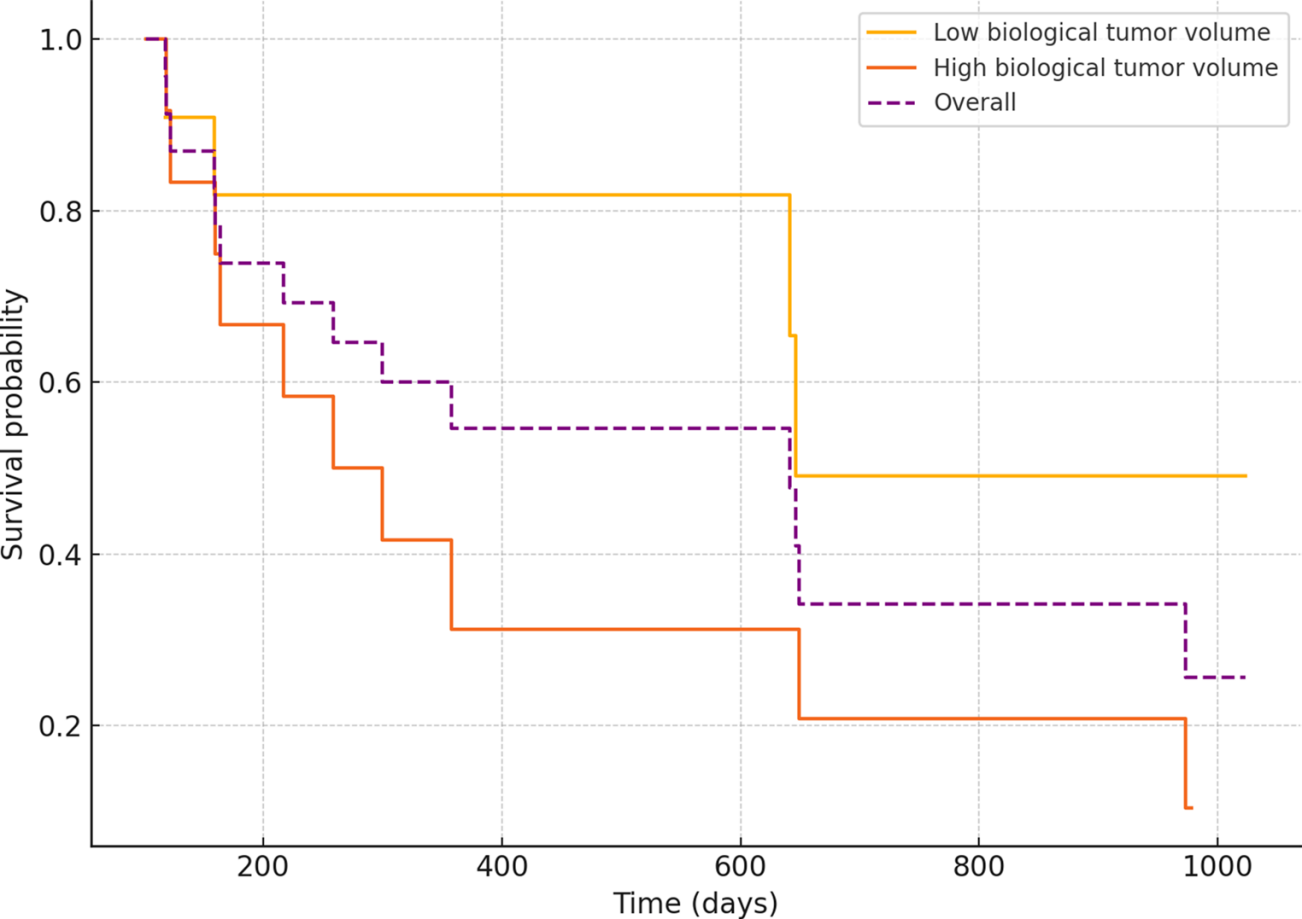
Kaplan-Meier survival curve illustrating the probability of overall survival in patients with recurrent high-grade glioma. The stepwise decline in survival probability reflects the observed survival durations in both groups. Censored observations are incorporated in the analysis. The dashed purple line represents the estimated survival function for the overall cohort. The orange line represents patients with a high biological tumor volume (defined as a tumor volume higher than the population’s median biological tumor volume). The yellow line represents patients with a low biological tumor volume (defined as a tumor volume equal to or lower than the population’s median biological tumor volume)

After diagnosis, two patients received adjuvant radiotherapy, one patient received a protein kinase inhibitor and twenty-three patients received an alkylating agent (nineteen patients received temozolomide and four received lomustine). A representative [^18^F]FET PET/MRI examination is provided in Fig. 3.

### Total amino-acid transport tumor volume is predictive of overall-survival in patients with recurrent high-grade glioma

The Cox proportional hazards regression model was assessed using the − 2 log likelihood statistic, which improved from 68.919 in the null model to 58.306 after inclusion of the predictors. The omnibus test of biological model coefficients indicated that the overall model reached statistical significance (χ² = 12.071, *p* = 0.002). The number of lesions was significantly associated with overall-survival (hazard ratio (HR) = 2.220, 95%-CI = 1.035–4.762, *p* = 0.041), showing that an increasing number of lesions visible on PET images was associated with a higher risk of mortality. In line with this, biological tumor volume was significantly associated with overall survival (HR = 1.032, 95%-CI: 1.002–1.064; *p* = 0.036), suggesting that larger biological tumor volumes were linked to a higher mortality risk (Table 2).

**Table 2:** Outcomes of the Cox proportional hazards model fit statistics (biological-clinical model)

Biological-clinical model		
Predictor	Tolerance	VIF
Biological tumor volume	0.620	1.613
Number of lesions	0.664	1.505
TBRmax ROI1	0.737	1.357
rCBVmax ROI1	0.814	1.228
Cho/NAA ratio	0.910	1.099
Treatment strategy	0.777	1.287
PET/MRI interaction model		
Predictor	**Tolerance**	**VIF**
TBRmax × rCBVmax	0.958	1.044
TBRmax × Cho/NAA ratio	0.007	149.838
rCBVmax × Cho/NAA ratio	0.007	150.353

The Cox proportional hazards regression model was assessed using the − 2 log likelihood statistic, which improved from 67.836 in the null model to 61.476 after inclusion of the predictors. The omnibus test of clinical model coefficients indicated that the overall model did not reach statistical significance (χ² = 6.031, *p* = 0.110). The TBRmax (*p* = 0.089) and rCBVmax-values (*p* = 0.088) and the Cho/NAA ratio (*p* = 0.734) were not significantly associated with overall-survival. Furthermore, treatment strategy after tumor recurrence diagnosis did not significantly impact overall-survival (HR = 0.208, *p* = 0.649) (Table 2). No strong correlations were observed between the independent variables, suggesting no major multicollinearity issues (Table 3).

**Table 3:** Multicollinearity diagnostics for main effects

Patient number Gender, age	Tumortype (WHO Grade)	TBRmax per laesion	rCBVmax per laesion	Cho/NAA ratio per laesion	Biological tumor volume (in ml)	Treatment after recurrence	Overall survival after PET/MRI (in days)
#1 F,69	Glioblastoma (WHO 4)	Laesion 1: 1.4 Laesion 2: 2.3 Laesion 3: 1.7	Laesion 1: 1.4 Laesion 2: 1.3 Laesion 3: 1.2	Laesion 1: 6.0 Laesion 2: 2.9 Laesion 3: X	50.64	Lomustine (alkylating agent)	119
#2 M,62	Glioblastoma (WHO 4)	Laesion 1: 5.0 Laesion 2: 4.2 Laesion 3: 1.1	Laesion 1: 1.2 Laesion 2: 1.3 Laesion 3: 1.3	Laesion 1: 1.1 Laesion 2: 2.1 Laesion 3: X	45.90	Lomustine (alkylating agent)	160
#3 M,63	Glioblastoma (WHO 4)	Laesion 1: 2.9	Laesion 1: 1.2	Laesion 1: 1.1	0.16	Adjuvant radiotherapy	646
#4 F, 67	Glioblastoma (WHO 4)	Laesion 1: 2.0	Laesion 1: 1.0	Laesion 1: 2.8	3.97	Third line temozolomide (alkylating agent)	641
#5 M, 74	Glioblastoma (WHO 4)	Laesion 1: 4.3	Laesion 1: 1.6	Laesion 1: 0.9	61.98	Third line temozolomide (alkylating agent)	259
#6 M, 45	Oligodendroglioma (WHO 3)	Laesion 1: 3.9 Laesion 2: 2.8 Laesion 3: 2.9	Laesion 1: 1.6 Laesion 2: 1.2 Laesion 3: 1.1	Laesion 1: 2.8 Laesion 2: 0.9 Laesion 3: 0.7	55.58	Regorafenib (protein kinase inhibitor)	164
#7 F, 77	Astrocytoma (WHO 3)	Laesion 1: 3.9	Laesion 1: 2.4	Laesion 1: 0.9	28.00	Third line temozolomide (alkylating agent)	649
#8 M, 62	Astrocytoma (WHO 3)	Laesion 1: 2.9	Laesion 1: 0.8	Laesion 1: 1.7	9.60	Third line temozolomide (alkylating agent)	N/A
#9 F, 34	Astrocytoma (WHO 4)	Laesion 1: 3.3 Laesion 2: 3.6 Laesion 3: 2.4	Laesion 1: 2.4 Laesion 2: 1.9 Laesion 3: 0.9	Laesion 1: 1.2 Laesion 2: X Laesion 3: X		Third line temozolomide (alkylating agent)	N/A
#10 F, 51	Glioblastoma (WHO 4)	Laesion 1: 2.2	Laesion 1: 2.1	Laesion 1: 1.3	7.48	Third line temozolomide (alkylating agent)	N/A
#11 M, 55	Glioblastoma (WHO 4)	Laesion 1: 3.3	Laesion 1: 1.4	Laesion 1: 0.9	42.30	Third line temozolomide (alkylating agent)	973
#12 M, 67	Glioblastoma (WHO 4)	Laesion 1: 2.2	Laesion 1: 1.3	Laesion 1: 0.7	1.08	Third line temozolomide (alkylating agent)	N/A
#13 M, 66	Glioblastoma (WHO 4)	Laesion 1: 3.6	Laesion 1: 1.7	Laesion 1: 2.5	69.77	Third line temozolomide (alkylating agent)	122
#14 M, 54	Astrocytoma (WHO 3)	Laesion 1: 5.0	Laesion 1: 1.2	Laesion 1: 6.0	50.10	Third line temozolomide (alkylating agent)	358
#15 M, 57	Glioblastoma (WHO 4)	Laesion 1: 3.8	Laesion 1: 1.4	Laesion 1: 1.0	45.73	Lomustin (alkylating agent)	217
#16 M, 50	Glioblastoma (WHO 4)	Laesion 1: 2.8	Laesion 1: 1.2	Laesion 1: 0.9	5.30	Lomustin (alkylating agent)	N/A
#17 M 60	Glioblastoma (WHO 4)	Laesion 1: 3.6	Laesion 1: 1.6	Laesion 1: 1.4	3.88	Lomustin (alkylating agent)	N/A
#18 F, 62	Glioblastoma (WHO 4)	Laesion 1: 2.1 Laesion 2: 2.0 Laesion 3: 2.2	Laesion 1: 1.4 Laesion 2: 2.3 Laesion 3: 1.4	Laesion 1: 1.2 Laesion 2: 1.3 Laesion 3: 1.0	34.95	Third line temozolomide (alkylating agent)	N/A
#19 F, 42	Glioblastoma (WHO 4)	Laesion 1: 2.3	Laesion 1: 1.3	Laesion 1: 0.8	5.94	Third line temozolomide (alkylating agent)	159
#20 F, 61	Glioblastoma (WHO 4)	Laesion 1: 4.1 Laesion 2: 4.3 Laesion 3: 3.2	Laesion 1: 1.5 Laesion 2: 1.3 Laesion 3: 0.7	Laesion 1: 0.7 Laesion 2: 0.6 Laesion 3: X	8.75	Adjuvant radiotherapy	118
#21 F, 35	Oligodendroglioma (WHO 3)	Laesion 1: 2.3 Laesion 2: 2.0	Laesion 1: 2.1 Laesion 2: 1.3	Laesion 1: 1.1 Laesion 2: X	3.00	Third line temozolomide (alkylating agent)	N/A
#22 M, 61	Glioblastoma (WHO 4)	Laesion 1: 4.3	Laesion 1: 1.1	Laesion 1: 1.1	21.60	Third line temozolomide (alkylating agent)	N/A
#23 M, 30	Astrocytoma (WHO 3)	Laesion 1: 6.6 Laesion 2: 2.6	Laesion 1: 2.6 Laesion 2: 1.1	Laesion 1: 0.9 Laesion 2: X	2.23	Third line temozolomide (alkylating agent)	N/A
#24 M, 45	Glioblastoma (WHO 4)	Laesion 1: 2.0	Laesion 1: 0.5	Laesion 1: 1.5	1.73	Adjuvant radiotherapy	N/A
#25 M, 51	Glioblastoma (WHO 4)	Laesion 1: 2.4	Laesion 1: 1.8	Laesion 1: 1.3	0.17	Third line temozolomide (alkylating agent)	N/A
#26 F, 36	Astrocytoma (WHO 4)	Laesion 1: 2.8 Laesion 1: 2.7	Laesion 1: 1.6 Laesion 2: 1.2	Laesion 1: 1.2 Laesion 2: X	21.10	Third line temozolomide (alkylating agent)	300

### Combined PET/MRI parameters did not enhance the prediction of overall-survival in patients with recurrent high-grade glioma

A Cox proportional hazards regression model was also applied to assess the relationship between PET/MRI derived interaction terms and overall-survival in patients with histopathologically confirmed tumor progression of high-grade glioma. The − 2 log likelihood statistic showed minimal improvement, decreasing from 67.836 in the null model to 66.334 after inclusion of the predictors. The overall model did not reach statistical significance (χ² = 1.502, *p* = 0.682), indicating that the interaction terms did not significantly contribute to survival prediction. None of the interaction terms were significantly associated with overall-survival: TBRmax × rCBVmax (*p* = 0.894), TBRmax × Cho/NAA ratio (*p* = 0.183), and rCBVmax × Cho/NAA ratio (*p* = 0.188). Severe multicollinearity was observed for two predictors: TBRmax × Cho/NAA ratio (VIF = 149.838) and rCBVmax × Cho/NAA ratio (VIF = 150.353), suggesting a near-linear dependency between these interaction terms and the original variables. Only the interaction term TBRmax × rCBVmax showed no multicollinearity (VIF = 1.044).

## Discussion

This study shows that total amino-acid transport tumor volume, rather than tumor-to-background ratio or MRI derived tumor features (i.e., rCBVmax and Cho/NAA-ratio), is predictive of overall-survival in high-grade glioma patients with tumor recurrence. This suggests that biological tumor volume, rather than solely the metabolic activity or perfusion of a single lesion, is a crucial factor in determining prognosis. This is consistent with previous studies in other tumor types, demonstrating that total tumor burden has a stronger impact on clinical outcome than focal tumor activity [15, 24–27]. However, considerable debate exists with regard to how such metabolic activity maps should be obtained. In the review of El-Galaly et al. various metabolic tumor volume segmentation methods that are used in malignant lymphoma, each method with its own benefits and shortcomings, are discussed [28]. They concluded that although most studies have used receiver operating curves to determine the optimal cut-off for metabolic tumor volume segmentation, most studies did not include a training-validation approach, leading to the risk of overestimation of the independent prognostic value of the metabolic tumor volume values [28–30]. The methodology used in this study to define biological tumor volume in recurrent high-grade glioma patients was based on existing evidence and guidelines [12, 18, 21, 22]. However, precautions still exist with regard to the interpretation of the results and the applied methodology. Albeit, as the more simple variable “number of lesions” was also found to be significantly correlated with overall-survival duration, it seems logical to assume that disease load in post-treatment high-grade glioma patients holds predictive value.

Additionally, the findings of the present study are in line with previous reports demonstrating the prognostic value of [^18^F]FET PET derived biological tumor volume in high-grade glioma patients. In the pretreatment setting, larger biological tumor volume has been shown to predict both progression-free survival [11, 13] and overall-survival [11, 13, 16, 31]. Similarly, in the post-treatment setting, [^18^F]FET PET derived biological tumor volume has also been reported a strong predictor for progression-free survival [13, 32] and overall-survival [12–14, 16, 18, 32] in high-grade glioma patients. With regard to the prognostic value of the biological tumor volume derived from [^18^F]FET at baseline, a recent review highlighted that [^18^F]FET PET can have a prognosticative role and predict survival in patients with gliomas and serves as a valuable tool to supplement the established clinical and histopathological parameters [33].One study in particular described how [^18^F]FET PET/MRI could be used to predict overall-survival in post-treatment high-grade glioma patients [18]. This study by Friedrich et al. investigated functional connectivity (FC) between metabolically active tumor regions and resting-state networks in 82 patients and found that tumor grade and isocitrate dehydrogenase mutation status were strong predictors of overall-survival (*p* < 0.001). Higher functional connectivity between the tumor and the dorsal attention network was associated with longer overall-survival (*p* < 0.01) and remained an independent predictor when adjusting for clinical factors, tumor volume, and the *MGMT* (O^6^-methylguanine-DNA methyltransferase) promoter methylation status (*p* < 0.05) [18]. In glioblastoma patients, tumor volume and functional connectivity with the visual network were independent survival predictors (*p* < 0.05). These findings suggest that functional connectivity between recurrent tumors and resting-state networks may serve as an additional prognostic imaging biomarker [18]. Our results are in agreement with the results of Friedrich et al., although the current investigation provides no insights with regard to the role of tumor location and functional connectivity. The observed severe multicollinearity between FET and PET parameters in the present study probably contributed to the lack of significant associations observed in the interaction model. Therefore, the added value of hybrid PET/MRI compared to stand-alone PET imaging in predicting overall survival in patients with recurrent high-grade glioma remains uncertain.

Other studies provided evidence that molecular imaging using different radiolabeled tracers at baseline holds predictive value in high-grade glioma patients. Singhal et al. reported on 102 histopathologically confirmed gliomas and showed that baseline TBRmean-values, derived from l-[methyl-(11)C]-methionine ([^11^C]methionine) PET/CT, could predict prognosis in gliomas. and was more accurate than [^18^F]−2-fluoro-2-deoxy-D-glucose ([^18^]FDG) PET and MRI in predicting survival in low-grade gliomas [33]. Also, Galldiks et al. showed that pretreatment biological tumor volume derived from [^11^C]methionine uptake, but not the semiquantitative [^11^C]methionine PET parameters (i.e., TBRmean, TBRmax and time to peak), could be a useful biologic prognostic marker in 40 anaplastic astrocytoma and glioblastoma patients [17]. Another study assessed the use of 3’-Deoxy-3’-[^18^F]Fluorothymidine ([^18^F]FLT) PET imaging in high-grade gliomas (*n* = 26) and demonstrated that proliferative volume, rather than SUVmax, was predictive of overall-survival. Among various segmentation methods, the signal-to-background ratio method showed the strongest association with overall-survival (*p* = 0.002) and effectively distinguished between short and long survival (*p* = 0.024) [18].

With regard to MRI derived parameters, our results conflict other studies. Two studied highlighted the use of rCBVmax to define tumor burden load and predict overall-survival in post-treatment glioblastoma [35, 36]. Comparison of current MRS results and other studies is hampered by the lack of studies using MRS in the post-treatment setting to predict overall-survival. In the pre-treatment setting, short echo time ¹H MRS has been explored in one study to predict progression-free survival and overall-survival. Machine learning models achieved approximately 82% accuracy in predicting overall-survival beyond 12 months [37].

To analyze larger imaging datasets more effectively, the role of Artificial Intelligence based approaches for the extraction of image features (e.g., radiomics) to predict overall-survival of high-grade glioma patients, is growing. One study applied machine learning to multiparametric MRI to identify radiomic features associated with early mortality (< 6 months) after gross total resection, with a Naïve Bayes classifier achieving the best performance (AUC = 0.769, accuracy = 80%), while a random survival forest model stratified patients into high- and low-risk groups [38]. Another study used a CNN-based model to classify glioblastoma patients into long- and short-term survivors with 74% accuracy [39]. A 2024 study analyzing T2-weighted and contrast-enhanced T1-weighted MRI data from 206 patients demonstrated that imaging-based models outperformed non-imaging models (AUC = 0.93 vs. 0.79, *p* = 0.038) in predicting survival at 8 months post-radiotherapy [40]. However, a systematic review by Garcia-Garcia et al. found that the radiomics quality score [41] of most studies was poor, preventing sound conclusions [42]. The review highlighted issues such as data availability, patient selection, and methodological heterogeneity, reaffirmed by another systematic review in 2025 [42, 43].

Taken together, these findings in the scientific literature highlight the potential of volume-based PET metrics over tumor-to-background measurements or MRI analyses (including radiomics approaches) for the prognostication of high-grade glioma patients. This increases the likelihood that the here presented results in post-treatment high-grade gliomas represent a biologically interpretable molecular imaging biomarker.

### Strengths and limitations

This retrospective study has several limitations. First, the sample size was relatively small, with only 26 patients included, which limits the statistical power of this study. Furthermore, the limited sample size prevents generalizability of the findings. Larger, multicenter cohorts are needed to validate the prognostic value of biological tumor volume in recurrent disease in high grade glioma patients. Furthermore, these studies need to assess the reproducibility of these findings across different imaging protocols. More specifically, further research should explore the potential added value of combined PET and MRI features using advanced machine learning techniques. Although, the prognostic value of functional connectivity, demonstrated in one prior study [18], was not assessed in this study, future research should explore the integration of functional imaging biomarkers with metabolic and structural parameters to refine survival prediction models.

## Conclusion

This study suggests that biological tumor volume and number of lesions, rather than tumor-to-background ratio or MRI-derived tumor features (i.e., rCBVmax and Cho/NAA ratio), are significant predictors of overall-survival in high-grade glioma patients with tumor recurrence. These findings suggest that tumor burden, rather than solely metabolic activity, perfusion or tissue disintegration at lesion level, plays a crucial role in prognosis. Future research should verify current findings in larger, prospective cohorts and should possibly integrate functional connectivity analyses.

## Electronic supplementary material

Below is the link to the electronic supplementary material.

Supplementary Material 1

## References

[1] Louis DN, Perry A, Wesseling P, Brat DJ, Cree IA, Figarella-Branger D, et al. The 2021 WHO classification of tumors of the central nervous system: a summary. Neuro Oncol. 2021;23:1231–51. 10.1093/neuonc/noab106. 34185076 10.1093/neuonc/noab106 PMC8328013

[2] Wen PY, Macdonald DR, Reardon DA, Cloughesy TF, Sorensen AG, Galanis E, et al. Updated response assessment criteria for high-grade gliomas: response assessment in neuro-oncology working group. J Clin Oncol. 2010;28:1963–72. 10.1200/JCO.2009.26.3541. 20231676 10.1200/JCO.2009.26.3541

[3] Carrete LR, Young JS, Cha S. Advanced imaging techniques for newly diagnosed and recurrent gliomas. Front Neurosci. 2022;16:787755. 35281485 10.3389/fnins.2022.787755 PMC8904563

[4] Thust SC, Van Den Bent MJ, Smits M. Pseudoprogression of brain tumors. J Magn Reson Imaging. 2018;48:571–89. 29734497 10.1002/jmri.26171 PMC6175399

[5] Maurer GD, Brucker DP, Stoffels G, Filipski K, Filss CP, Mottaghy FM, et al. 18F-FET PET imaging in differentiating glioma progression from treatment-related changes: a single-center experience. J Nucl Med. 2020;61:505–11. 31519802 10.2967/jnumed.119.234757

[6] Langen KJ, Galldiks N, Mauler J, Kocher M, Filß CP, Stoffels G, et al. Hybrid PET/MRI in cerebral glioma: current status and perspectives. Cancers (Basel). 2023;15:3577. 10.3390/cancers15143577. 37509252 10.3390/cancers15143577 PMC10377176

[7] Van de Weijer T, Broen MPG, Moonen RPM, Hoeben A, Anten M, Hovinga K, et al. The use of (18)F-FET-PET-MRI in Neuro-Oncology: the best of both Worlds—A. Narrative Rev Diagnostics (Basel). 2022;12:1205. 10.3390/diagnostics12051202. 10.3390/diagnostics12051202 PMC9140561 35626357

[8] Suchorska B, Jansen NL, Linn J, Kretzschmar H, Janssen H, Eigenbrod S, et al. Biological tumor volume in 18FET-PET before radiochemotherapy correlates with survival in GBM. Neurology. 2015;84:710–9. 10.1212/WNL.0000000000001262. 25609769 10.1212/WNL.0000000000001262

[9] Gutsche R, Lowis C, Ziemons K, Kocher M, Ceccon G, Regio Brambilla C, et al. Automated brain tumor detection and segmentation for treatment response assessment using amino acid PET. J Nucl Med. 2023;64:1594–602. 10.2967/jnumed.123.265725. 37562802 10.2967/jnumed.123.265725

[10] Ceccon G, Lohmann P, Werner JM, Tscherpel C, Dunkl V, Stoffels G, et al. Early treatment response assessment using (18)F-FET PET compared with Contrast-Enhanced MRI in glioma patients after adjuvant Temozolomide chemotherapy. J Nucl Med. 2021;62:918–25. 10.2967/jnumed.120.254243. 33158907 10.2967/jnumed.120.254243

[11] Wan Q, Lindsay C, Zhang C, Kim J, Chen X, Li J, et al. Comparative analysis of deep learning and radiomic signatures for overall survival prediction in recurrent high-grade glioma treated with immunotherapy. Cancer Imaging. 2025;25:5. 10.1186/s40644-024-00818-0. 39838503 10.1186/s40644-024-00818-0 PMC11752626

[12] Hansen ST, Jacobsen KS, Kofoed MS, Petersen JK, Boldt HB, Dahlrot RH, et al. Prognostic factors to predict postoperative survival in patients with recurrent glioblastoma. Nucl Med. 2024;23:100308. 10.1016/j.wnsx.2024.100308. 10.1016/j.wnsx.2024.100308 PMC10997900 38584878

[13] Friedrich M, Werner JM, Steinbach JP, Sabel M, Herrlinger U, Piroth M, et al. Functional connectivity between tumor region and resting-state networks as imaging biomarker for overall survival in recurrent gliomas diagnosed by O-(2-[(18)F]fluoroethyl)-l-tyrosine PET. Neurooncol. Adv. 2025;7:vdaf023. 10.1093/noajnl/vdaf023. 10.1093/noajnl/vdaf023 PMC11904474 40084168

[14] Langen K-J, Bartenstein P, Boecker H, Brust P, Coenen H, Drzezga A, et al. PET-und SPECT-Untersuchungen von Hirntumoren Mit Radioaktiv markierten aminosäuren. Nuklearmed. 2011;50:167–73. 10.3413/nuk-2011041 21789338

[15] Gallicchio R, Nardelli A, Venetucci A, Capacchione D, Pelagalli A, Sirignano C, et al. F-18 FDG PET/CT metabolic tumor volume predicts overall survival in patients with disseminated epithelial ovarian cancer. Eur J Radiol. 2017;93:107–13. 10.1016/j.ejrad.2017.05.036. 28668403 10.1016/j.ejrad.2017.05.036

[16] Waltenberger M, Furkel J, Rohrich M, Salome P, Debus C, Tawk B, et al. The impact of tumor metabolic activity assessed by (18)F-FET amino acid PET imaging in particle radiotherapy of high-grade glioma patients. Front Oncol. 2022;12:901390. 10.3389/fonc.2022.901390. 36203443 10.3389/fonc.2022.901390 PMC9531169

[17] Galldiks N, Dunkl V, Kracht LW, Vollmar S, Jacobs AH, Fink GR, et al. Volumetry of [¹¹C]-methionine positron emission tomographic uptake as a prognostic marker before treatment of patients with malignant glioma. Mol Imaging. 2012;11:516–27. 23084252

[18] Idema AJ, Hoffmann AL, Boogaarts HD, Troost EG, Wesseling P, Heerschap A, et al. 3’-Deoxy-3’-18F-fluorothymidine PET-derived proliferative volume predicts overall survival in high-grade glioma patients. J Nucl Med. 2012;53:1904–10. 10.2967/jnumed.112.105544.18. 23077112 10.2967/jnumed.112.105544

[19] Floeth FW, Pauleit D, Sabel M, Reifenberger G, Stoffels G, Stummer W, et al. 18F-FET PET differentiation of ring-enhancing brain lesions. J Nucl Med. 2006;47:776–82. 16644747

[20] Hutterer M, Nowosielski M, Putzer D, Jansen NL, Seiz M, Schocke M, et al. [18F]-fluoro-ethyl-L-tyrosine PET: a valuable diagnostic tool in neuro-oncology, but not all that glitters is glioma. Neuro Oncol. 2013;15:341–51. 23335162 10.1093/neuonc/nos300 PMC3578481

[21] Albert NL, Galldiks N, Ellingson BM, van den Bent MJ, Chang SM, Cicone F, et al. PET-based response assessment criteria for diffuse gliomas (PET RANO 1.0): a report of the RANO group. Lancet Oncol. 2024;25:e29–41. 10.1016/S1470-2045(23)00525-9. 38181810 10.1016/S1470-2045(23)00525-9 PMC11787868

[22] Pauleit D, Floeth F, Tellmann L, Hamacher K, Hautzel H, Müller HW, et al. Comparison of O-(2-18F-fluoroethyl)-L-tyrosine PET and 3-123I-iodo-alpha-methyl-L-tyrosine SPECT in brain tumors. J Nucl Med. 2004;45:374–81. 15001676

[23] R Core Team. R: A Language and Environment for Statistical Computing. Vienna, Austria: R Foundation for Statistical Computing. 2010. Available from: https://www.R-project.org/

[24] Lin CY, Chang YC, Wang IT, Hsieh MH, Wang CW, Lin SM, et al. Metabolic tumor volume predicts overall survival in patients with primary pulmonary lymphoepithelioma-like carcinoma. Oncol Lett. 2019;18:6143–9. 10.3892/ol.2019.10954. 31788088 10.3892/ol.2019.10954 PMC6864931

[25] Liu L, Jin F, Fan H. Metabolic tumor volume and the survival of patients with Non-Hodgkin lymphoma treated with chimeric antigen receptor T cell therapy: a meta-analysis. Front Immunol. 2024;15:1433012. 10.3389/fimmu.2024.1433012. 39267739 10.3389/fimmu.2024.1433012 PMC11390410

[26] Rijo-Cedeño J, Mucientes J, Álvarez O, Royuela A, Seijas Marcos S, Romero J, et al. Metabolic tumor volume and total lesion Glycolysis as prognostic factors in head and neck cancer: systematic review and meta-analysis. Head Neck. 2020;42:3744–54. 10.1002/hed.26461. 32914474 10.1002/hed.26461

[27] Evangelista L, Urso L, Caracciolo M, Stracuzzi F, Panareo S, Cistaro A, et al. FDG PET/CT Volume-Based quantitative data and survival analysis in breast Cancer patients: A systematic review of the literature. Curr Med Imaging. 2023;19:807–16. 10.2174/1573405618666220329094423. 35352652 10.2174/1573405618666220329094423

[28] El-Galaly TC, Villa D, Cheah CY, Gormsen LC. Pre-treatment total metabolic tumour volumes in lymphoma: does quantity matter? Br J Haematol. 2022;197:139–55. 10.1111/bjh.18016. 35037240 10.1111/bjh.18016

[29] Boellaard R, Buvat I, Nioche C, Ceriani L, Cottereau AS, Guerra L, et al. International benchmark for total metabolic tumor volume measurement in baseline (18)F-FDG PET/CT of lymphoma patients: A milestone toward clinical implementation. J Nucl Med. 2024;65:1343–8. 10.2967/jnumed.124.267789. 39089812 10.2967/jnumed.124.267789 PMC11372260

[30] Boellaard R, Zwezerijnen GJC, Buvat I, Champion L, Hovhannisyan-Baghdasarian N, Orlhac F, et al. Measuring total metabolic tumor volume from (18)F-FDG PET: A reality check. J Nucl Med. 2025. 10.2967/jnumed.124.269271. (Epub ahead of print). 40081961 10.2967/jnumed.124.269271

[31] Mittlmeier LM, Suchorska B, Ruf V, Holzgreve A, Brendel M, Herms J, et al. (18)F-FET PET uptake characteristics of Long-Term IDH-Wildtype diffuse glioma survivors. Cancers (Basel). 2021;13:3163. 10.3390/cancers13133163. 34202726 10.3390/cancers13133163 PMC8268019

[32] Carles M, Popp I, Starke MM, Mix M, Urbach H, Schimek-Jasch T, et al. FET-PET radiomics in recurrent glioblastoma: prognostic value for outcome after re-irradiation? Radiat Oncol. 2021;16:46. 10.1186/s13014-020-01744-8. 33658069 10.1186/s13014-020-01744-8 PMC7931514

[33] Muoio B, Giovanella L, Treglia G. Recent developments of 18F-FET PET in Neuro-oncology. Curr Med Chem. 2018;25:3061–73. 10.2174/0929867325666171123202644. 29173147 10.2174/0929867325666171123202644

[34] Singhal T, Narayanan TK, Jacobs MP, Bal C, Mantil JC. 11 C-methionine PET for grading and prognostication in gliomas: a comparison study with 18F-FDG PET and contrast enhancement on MRI. J Nucl Med. 2012;53:1709–15. 10.2967/jnumed.111.102533. 23055534 10.2967/jnumed.111.102533

[35] Hu LS, Eschbacher JM, Heiserman JE, Dueck AC, Shapiro WR, Liu S, et al. Reevaluating the imaging definition of tumor progression: perfusion MRI quantifies recurrent glioblastoma tumor fraction, pseudoprogression, and radiation necrosis to predict survival. Neuro Oncol. 2012;14:919–30. 10.1093/neuonc/nos112. 22561797 10.1093/neuonc/nos112 PMC3379799

[36] Fong C, Parpia S, Yemen B, Tsai S, Greenspoon J. Using magnetic resonance perfusion to stratify overall survival in treated High-Grade gliomas. Can J Neurol Sci. 2019;46:533–9. 10.1017/cjn.2019.225. 31284880 10.1017/cjn.2019.225

[37] Sacli-Bilmez B, Danyeli AE, Yakicier MC, Aras FK, Pamir MN, Özduman K, et al. Magnetic resonance spectroscopic correlates of progression free and overall survival in glioblastoma, IDH-wildtype, WHO grade-4. Front Neurosci. 2023;17:1149292. 10.3389/fnins.2023.1149292. 37457011 10.3389/fnins.2023.1149292 PMC10339315

[38] Cepeda S, Pérez-Nuñez A, García-García S, García-Pérez D, Arrese I, Jiménez-Roldán L, et al. Predicting Short-Term survival after gross total or near total resection in glioblastomas by machine Learning-Based radiomic analysis of preoperative MRI. Cancers (Basel). 2021;13. 10.3390/cancers13205047. 10.3390/cancers13205047 PMC8533879 34680199

[39] Ben Ahmed K, Hall LO, Goldgof DB, Gatenby R. Ensembles of convolutional neural networks for survival time Estimation of High-Grade glioma patients from multimodal MRI. Diagnostics (Basel). 2022;12. 10.3390/diagnostics12020345. 10.3390/diagnostics12020345 PMC8871067 35204436

[40] Chelliah A, Wood DA, Canas LS, Shuaib H, Currie S, Fatania K, et al. Glioblastoma and radiotherapy: A multicenter AI study for survival predictions from MRI (GRASP study). Neurooncology. 2024;26:1138–51. 10.1093/neuonc/noae017. 10.1093/neuonc/noae017 PMC11145448 38285679

[41] Lambin P, Leijenaar RTH, Deist TM, Peerlings J, de Jong EEC, van Timmeren J, et al. Radiomics: the Bridge between medical imaging and personalized medicine. Nat Rev Clin Oncol. 2017;14:749–62. 10.1038/nrclinonc.2017.141. 28975929 10.1038/nrclinonc.2017.141

[42] Garcia-Garcia S, Garcia-Galindo M, Arrese I, Sarabia R, Cepeda S. Current evidence, limitations and future challenges of survival prediction for glioblastoma based on advanced noninvasive methods: A narrative review. Med (Kaunas). 2022;58. 10.3390/medicina58121746. 10.3390/medicina58121746 PMC9786785 36556948

[43] Awuah WA, Ben-Jaafar A, Roy S, Nkrumah-Boateng PA, Tan JK, Abdul-Rahman T, et al. Predicting survival in malignant glioma using artificial intelligence. Eur J Med Res. 2025;30:61. 10.1186/s40001-025-02339-3. 39891313 10.1186/s40001-025-02339-3 PMC11783879

