# Supplementary material for: Biological tumor volume predicts survival in recurrent High-Grade glioma: A multiparametric [18F]FET PET/MRI study
Source: Eur J Nucl Med Mol Imaging. 2025 Jul 24;53(2):833–42. doi: 10.1007/s00259-025-07469-8 (PMC12830414; doi:10.1007/s00259-025-07469-8)
Supplement: Supplementary file 1 — Supplementary Material 1 [file 259_2025_7469_MOESM1_ESM.docx]

| Pulse Sequence | Parameters |
| --- | --- |
| CE-T1WI |  |
| Repetition Time (ms) | 2400 |
| Echo Time (ms) | 2.26 |
| Flip angle (degrees) | 8.0 |
| Section thickness (mm) | 1.00 |
| FLAIR |  |
| Repetition Time (ms) | 5000 |
| Echo Time (ms) | 385 |
| Inversion Time (ms) | 1600 |
| Section thickness (mm) | 0.9 |
| T2WI |  |
| Repetition Time (ms) | 6000 |
| Echo Time (ms) | 100.0 |
| Flip angle (degrees) | 120.0 |
| Section thickness (mm) | 3.0 |
| DSC |  |
| Repetition Time (ms) | 2250 |
| Echo Time (ms) | 28.0 |
| Flip angle (degrees) | 90.0 |
| Section thickness (mm) | 4.0 |
| Pre-bolus used | Yes |
| Standard dose (mmol/kg) | 0.1 mmol/kg |
| ^1^H MRS |  |
| Repetition Time (ms) | 2000 |
| Echo Time (ms) | 30.0 |
| Flip angle (degrees) | 90.0 |
| Voxel size (mm) | 20 × 20 × 20 |
| Spectral width (Hz) | 1200 |
| Water suppression | Yes |

***Supplementary Table 1 Details on the MR imaging acquisition protocol***

CE-T1WI = contrast enhanced T1 weighted imaging, DSC = dynamic susceptibility contrast imaging, FLAIR = fluid attenuated inversion recovery, ^1^H MRS = proton magnetic resonance spectroscopy, T2WI = T2 weighted imaging
